# Management of proximal femur malunion and distal femur nonunion via proximal femoral nailing and free fibular graft: A case report

**DOI:** 10.1016/j.ijscr.2023.108979

**Published:** 2023-10-24

**Authors:** Seyyed Hadi Kalantar, Nima Bagheri, Shahabaldin Beheshti Fard, Sina Afzal

**Affiliations:** a Joint Reconstruction Research Center, Tehran University of Medical Sciences, Tehran, Iran; b Department of Orthopedic Surgery, School of Medicine, Shahid Beheshti University of Medical Sciences, Tehran, Iran

**Keywords:** Nonunion, Malunion, Distraction osteogenesis, Bone graft, Case report

## Abstract

**Introduction and importance:**

Concurrent ipsilateral femoral malunion and nonunion present substantial clinical challenges requiring comprehensive surgical interventions. We describe a unique case of a 65-year-old male with these complications who was treated with a proximal femoral osteotomy, radical sequestrectomy, and free fibula graft.

**Case presentation:**

The patient underwent over 10 years of multiple surgical interventions, including hardware removal, local debridement, antibiotic-loaded cement spacer placement, autologous bone grafting, and external fixator applications, yet infectious non-union persisted. Additionally, a periprosthetic subtrochanteric fracture led to malunion due to his lack of consent for surgery. Despite attempted distraction osteogenesis, limited patient cooperation hindered success. Subsequent Free fibula grafting ultimately achieved satisfactory union, enabling full weight-bearing and according to the Short Form-36 (SF-36), the patient's physical function score increased from 30 % to 65 %.

**Clinical discussion:**

In the field of orthopedic surgery, addressing infectious non-union in long bones presents a notable clinical challenge. Radical debridement is fundamental to its management, a procedure that, in severe and resistant cases, may give rise to critical-sized bone defects.

To address these defects, a spectrum of biological reconstruction techniques has evolved over time. The selection of the most appropriate strategy necessitates individualization based on the patient and the specific nonunion characteristics.

**Conclusion:**

This case underscores the importance of radical debridement for infectious non-union. It emphasizes the consideration of biological reconstruction for critical-sized defects, particularly when concurrent deformities are present. Patient compliance is pivotal for treatment success, necessitating alternative approaches when cooperation is compromised.

## Highlights

• One of the fundamental principles in treating infectious non-unions of long bones is the need for thorough and extensive debridement.
• Distraction osteogenesis and cortical strut grafting are two commonly used methods for the biological reconstruction of critical sized bone defects.

## 1 Introduction

Concurrent ipsilateral malunion and nonunion of the femur represent challenging clinical scenarios, often necessitating complex surgical interventions. Proximal femur malunion can result in deformity, pain, and functional impairment, while distal femur nonunion poses a risk of persistent instability and compromised limb function [1]. The concurrent occurrence of these conditions within the same limb further complicates management, demanding a multifaceted approach that addresses both the deformity and the nonunion [2].

Surgical treatment options for proximal femur malunion typically involve osteotomy and internal fixation to correct the malalignment. In contrast, addressing distal femur infectious nonunion often requires radical debridement of infected bone resulting in bone defect. The simultaneous presence of these conditions necessitates a meticulous surgical plan that achieves infection eradication, stability, alignment, and union while minimizing morbidity. Proximal femoral nail (PFN) can provide good stability from the proximal to the distal femur [3].

On a contrasting note, certain treatment modalities employed to address the complexities of such cases, such as distraction osteogenesis, demand a high level of patient cooperation and compliance [4]. It is crucial to underscore that the success of these treatments is profoundly contingent on the patient's active engagement and adherence to the prescribed protocols; otherwise, the efficacy of these interventions may be compromised [5].

This case report elucidates the treatment considerations and formidable challenges encountered in managing a patient characterized by low adherence and cooperation levels, coupled with the concurrent presentation of malunion in the subtrochanteric region and a decade-long history of infectious non-union in the distal femoral shaft. To the best of our knowledge, an extensive review of the literature has revealed no parallel cases resembling the unique challenges and clinical complexities presented in this report.

This study has been reported in line with the SCARE criteria [6].

## 2 Case presentation

### 2.1 Clinical history

A 61-year-old male presented with a complex history of a traumatic open fracture of the distal shaft of the left femur sustained in a pedestrian-car accident 13 years prior. The patient had no underlying medical conditions but had a history of opioid addiction.

**Fig. 1:**
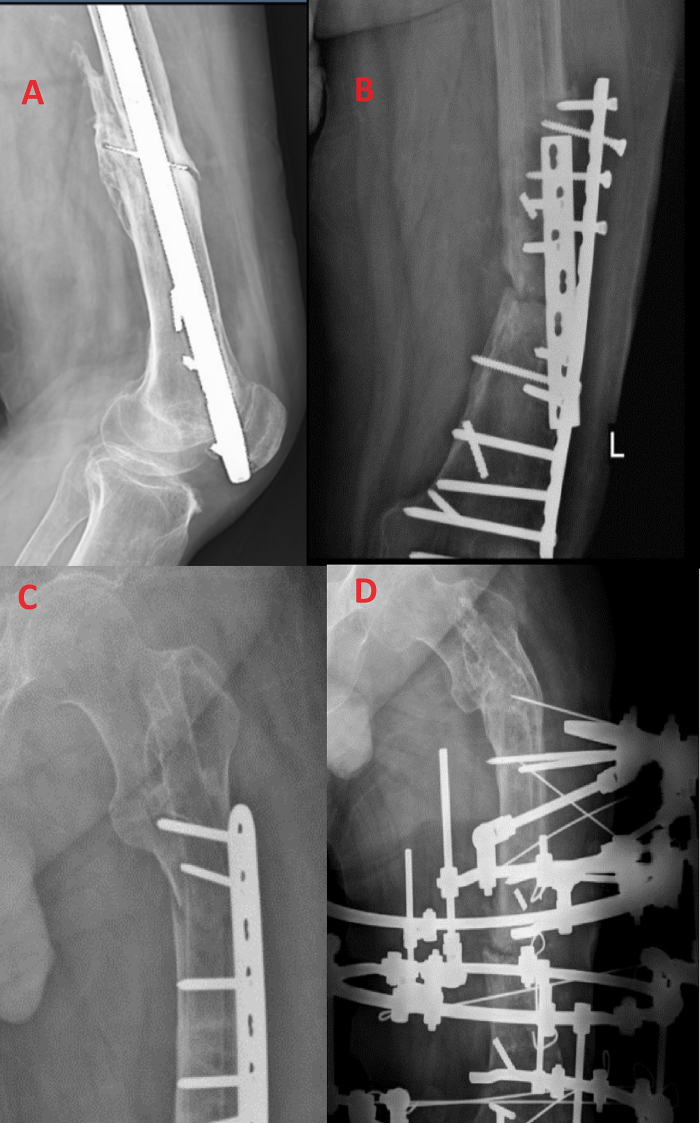
The progression of complications before referral to our center. **(A)** Infectious non-union and device failure resulting in device intrusion into the knee joint. **(B)** Recalcitrant non-union and subsequent failure of successive devices employed. **(C)** Periprosthetic subtrochanteric fracture following a patient fall. **(D)** Removal of all prior internal devices and the application of an Ilizarov fixator. Malunion of the previous periprosthetic fracture is evident in the subtrochanteric region.

The initial management at the primary center involved intramedullary femoral nail implantation. Subsequently, the patient experienced a series of recurrent infectious non-union complications attributed to the Acinetobacter Baumann pathogen, prompting a range of interventions:

1. One year post-initial surgery, the patient was indicated for surgery due to an infected non-union. However, this procedure was deferred due to the patient's lack of consent.
2. Six years after the initial surgery, the patient presented with an inability to flex the knee due to the intra-articular entry of the femoral nail. The patient consented to surgery, which consisted of removal of the femoral nail, local debridement, femoral osteoplasty, demineralized bone matrix implantation, and compression using a locking compression plate (LCP).
3. Eleven months later, the patient presented with a recurrence of infectious non-union and device failure. Surgical intervention included removal of previous devices, temporary antibiotic-loaded cement spacer placement at the non-union site, and the insertion of a longer plate.
4. Two months following the above surgery: spacer removal and iliac crest bone graft implantation were performed.
5. Six months later, the patient sustained a periprosthetic fracture in the subtrochanteric region due to a fall. Non-operative management was chosen due to the patient's reluctance for additional surgery, involving skin traction and bed rest.
6. One year after the fall, a fistula developed at the non-union site, accompanied by severe discharge. Surgical treatment involved device removal, local debridement, and the application of an Ilizarov external fixator. Notably, X-rays during this phase revealed malunion of the previous subtrochanteric fracture [Fig. 1].

Subsequently, the patient was referred to our center due to the persistent non-union and Ilizarov pin tract infection.

### 2.2 Surgical technique

**Fig. 2:**
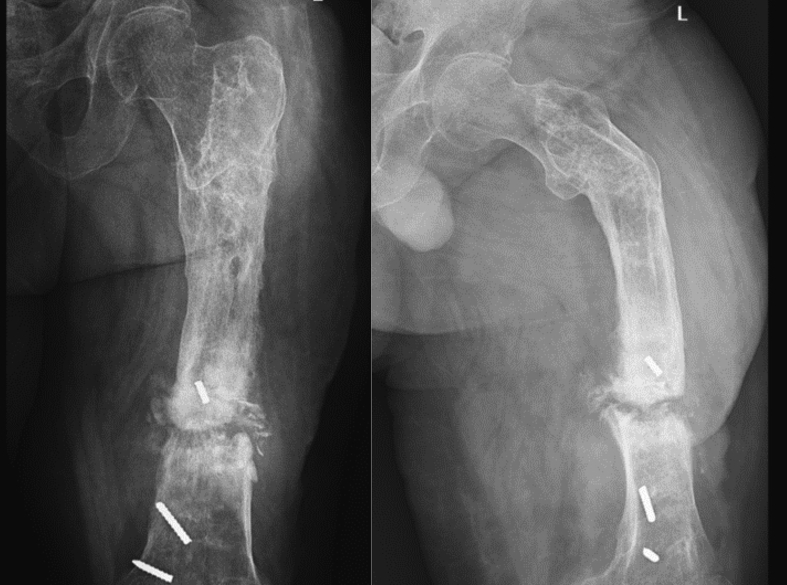
Anteroposterior and lateral views of the femur following Ilizarov fixator removal and prior to distal femoral shaft sequestrectomy and subtrochanteric corrective osteotomy.

**Fig. 3:**
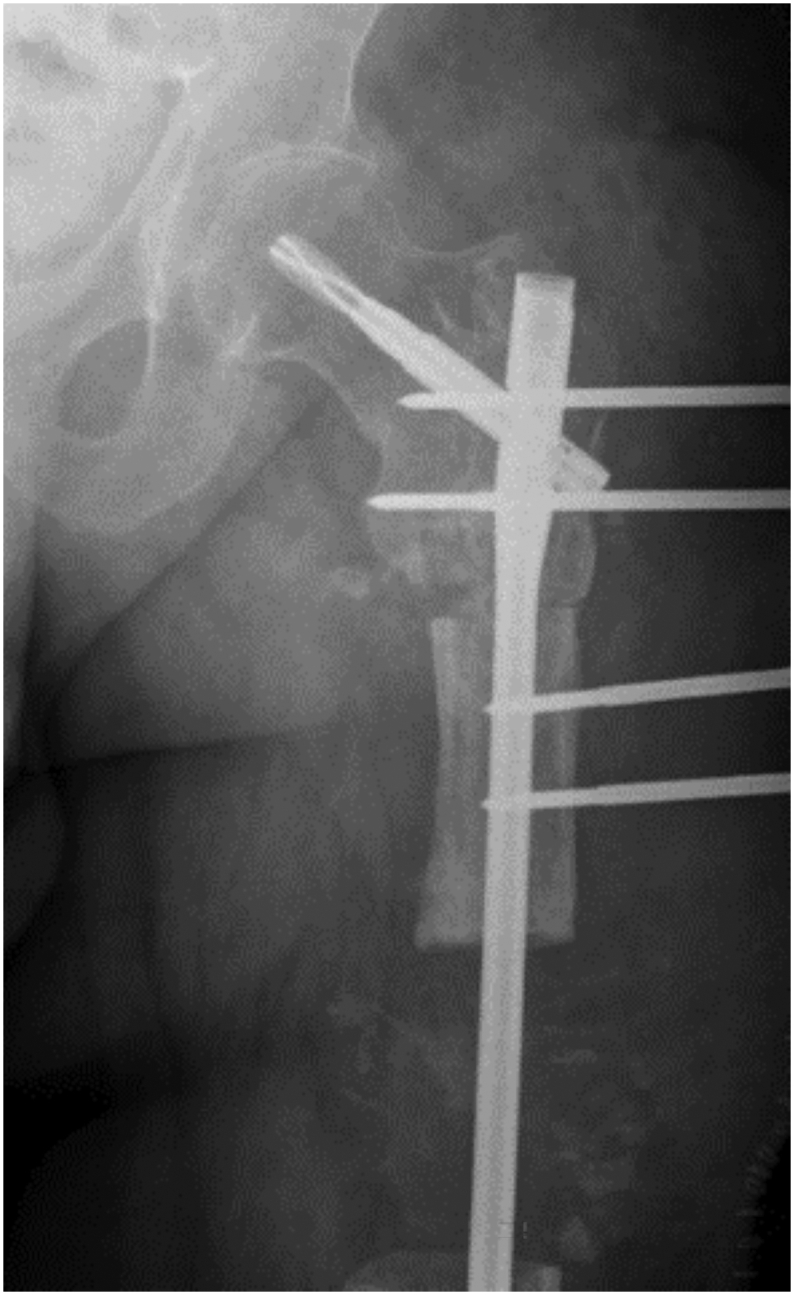
Simultaneous sequestrectomy of the distal femoral shaft and corrective osteotomy of the subtrochanteric region, accompanied by the application of a Hoffmann external fixator to initiate distraction osteogenesis.

Upon referral, we employed a two-stage management strategy for the patient. In the initial stage, Ilizarov external fixation was removed, and antibiotic therapy was administered until normalization of erythrocyte sedimentation rate (ESR) and C-reactive protein (CRP) levels [Fig. 2]. Given the presence of concurrent malunion in the subtrochanteric region and persistent infectious non-union in the distal femur, a decision was made for radical sequestrectomy in the non-union region, corrective osteotomy in the subtrochanteric region, and simultaneous distraction osteogenesis of the intercalary segment using a Hoffman's external fixator over a Long-gamma PFN. The radical sequestrectomy was performed at the nonunion site, creating a 10 cm defect. The paprika sign was used to identify the border between the sequestrum and normal bone [Fig. 3].

**Fig. 4:**
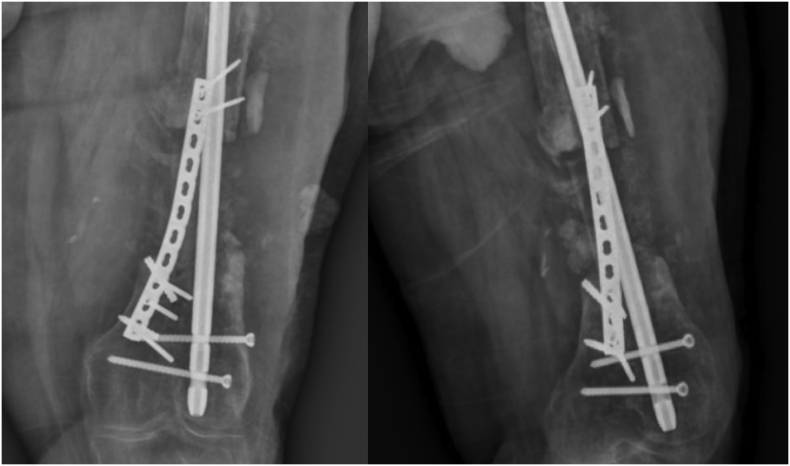
Postoperative radiograph following free fibular graft implantation.

Unfortunately, due to the patient's limited cooperation and non-adherence to external fixator adjustment instructions, and follow-up visits, after 4 cm of distraction, premature consolidation occurred in the distraction zone. Given the proper alignment of the union in the proximal femur and a critical-sized bone defect, the decision was made to employ a free bone graft from the ipsilateral fibula. The procedure involved a medial femur approach to prepare the defect site, followed by a lateral approach for proximal and distal osteotomy of the fibula. A 10-cm fibula graft was harvested and transplanted to the defect site and secured with a pre-contoured reconstruction plate. Subsequently, autologous cancellous bone graft from the ilium was utilized to fill the surrounding the graft [Fig. 4].

### 2.3 Follow-up and outcome

Postoperatively, the patient's limb was immobilized in a long leg splint for three weeks. Hip and knee physiotherapy was initiated at week three to strengthen the abductor and quadriceps muscles and improve knee range of motion (ROM). patient's knee ROM after completing the rehabilitation courses was 0 to 90 degrees.

**Fig. 5:**
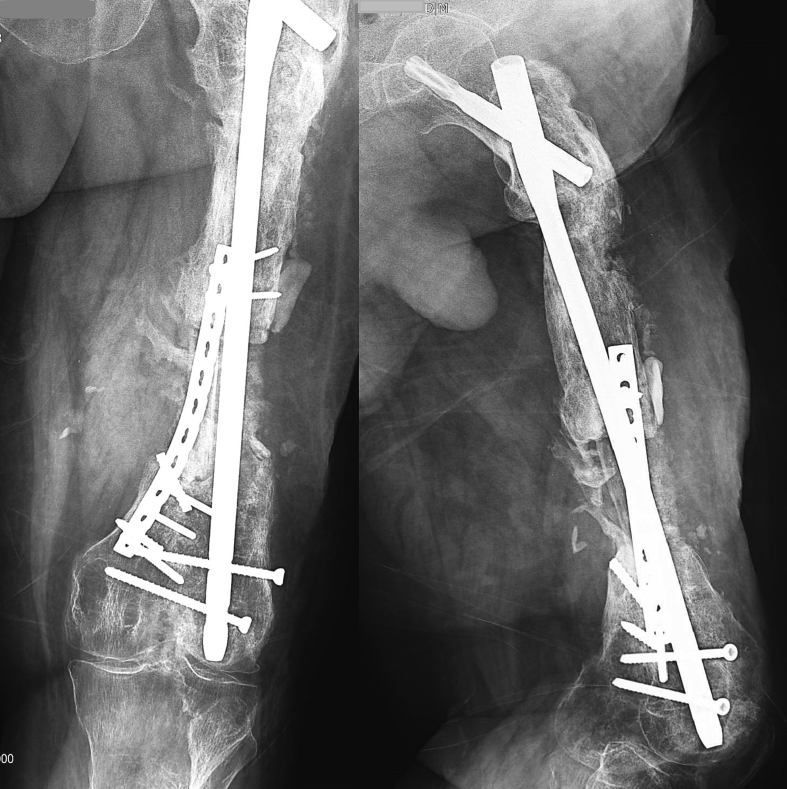
Patient's radiograph 9 months after free fibula bone graft from medial approach.

Femur imaging was conducted at three-month intervals to monitor the union progress. After three months, partial weight-bearing was initiated, and at nine months, with acceptable union, full weight-bearing was permitted with support of a cane [Fig. 5].

The patient had a 1-cm leg length discrepancy, which was managed with a shoe lift. The patient had severe muscle weakness and a slight limp due to long-term non-weightbearing and multiple surgeries. The achieved union also was not as strong as the intact bone. Therefore, they were advised to always use a cane. According to the Short Form-36 (SF-36), the patient's physical function score increased from 30 % to 65 %.

## 3 Discussion

Infectious non-union of long bones presents a substantial challenge for orthopedic surgeons, necessitating a meticulous and comprehensive treatment approach. Over time, many different treatment methods have been developed for infectious non-union [7,8]. The two-stage approach is now considered the preferred strategy. This involves first eliminating the infection, followed by achieving bone union in the second stage. The success of the first stage depends on the complete removal of all infected and unviable soft and bone tissues [9].

In the case presented here, the patient had a history of multiple surgeries employing various techniques and strategies, all characterized by localized and limited debridement. Despite these efforts, infectious non-union persisted, underscoring the critical importance of radical debridement in the initial stage of treatment [10]. Upon referral to our institution, we recognized the imperative for extensive debridement, resulting in a critical-sized bone defect within the femur.

The second stage of treatment then commenced with the goal of achieving union, which in this case required biological reconstruction due to the defect size [11]. Two possible approaches could be employed in such cases: distraction osteogenesis via intercalary segment transportation or free bone grafting [12]. In our unique case, characterized by concurrent malunion in the proximal femur, we opted for distraction osteogenesis, allowing us to simultaneously address both challenges through proximal corrective osteotomy.

However, it is noteworthy that despite our therapeutic intentions, the patient's limited cooperation and adherence to instructions adversely led to premature consolidation of the distraction osteogenesis process, necessitating its discontinuation [13]. Premature consolidation of bone fractures, a common complication in children and patients with low cooperation, is caused by inadequate distraction of the fracture fragments [14].

Allsopp et al.'s systematic review found no significant difference in union rates between vascularized and non-vascularized bone grafts. However, in patients requiring multiple surgeries, extensive fibrosis can make microsurgery infeasible. Therefore, non-vascularized bone grafting was chosen as the treatment strategy for this patient [15]. The fibula bone was selected for this purpose. Remarkably, this procedure was performed approximately 10 years after the initial trauma, illustrating the protracted and complex nature of the patient's medical history.

This case underscores the formidable challenges posed by infectious non-union in long bones, emphasizing the pivotal role of extensive debridement in infection eradication. It also highlights the importance of considering both biological reconstruction options in cases with substantial bone defects, along with the need for patient compliance to optimize outcomes in distraction osteogenesis. This study demonstrates that it is possible to successfully manage two fracture complications in a patient with poor cooperation.

## 4 Conclusion

Radical debridement of infected and necrotic soft tissue and bone stands as a foundational and imperative measure in the management of infected non-union, particularly in cases that have persisted over the long term. The extensive debridement process may, in some instances, result in the creation of a critical-sized bone defect, thereby necessitating subsequent biological bone reconstruction.

When faced with such critical-sized defects, two primary options emerge: distraction osteogenesis and free bone grafts. In situations where there is a concurrent deformity at the opposite end of the bone, a corrective osteotomy can be performed to not only create an intercalary segment but also restore proper bone alignment.

It is pivotal to underscore that the success of these treatment modalities is profoundly contingent on the patient's active participation and commitment to the prescribed treatment plan. In this context, patient cooperation assumes paramount significance. In cases where cooperation and adherence to treatment are compromised, alternative approaches, such as free bone grafting, may need to be contemplated.

## Abbreviations

PFN: proximal femoral nail
LCP: locking compression plate
ESR: erythrocyte sedimentation rate
CRP: C-reactive protein
ROM: range of motion

## Consent for publication

Written informed consent was obtained from the patient for publication and any accompanying images. A copy of the written consent is available for review by the Editor-in-Chief of this journal on request.

## Ethics approval

The Ethics Committee of the Tehran University of Medical Sciences, School of Medicine, Tehran, Iran, does not require ethics review for case reports of medical procedures performed within the normal scope of care, if written consent is obtained from the individual patient.

## Funding

None.

## Author contribution

**SHK** and **NB** conceptualized the study. **SA** drafted the initial manuscript. **SBF** collected the patient's data. All the authors read and approved the final version of manuscript.

## Guarantor

The Guarantor is: **Sina Afzal**.

## Conflict of interest statement

The authors declare that they have no competing interests.

## Data Availability

The material presented in this study is available from the corresponding author on a reasonable request.
